# Routine Head Computed Tomography for Patients in the Emergency Room with Trauma Requires Both Thick- and Thin-Slice Images

**DOI:** 10.1155/2016/5781790

**Published:** 2016-02-11

**Authors:** Kazuhide Maetani, Jun Namiki, Shokei Matsumoto, Katsutoshi Matsunami, Atsushi Narumi, Toshimi Tsuneyoshi, Masanobu Kishikawa

**Affiliations:** ^1^Department of Emergency and Critical Care Medicine, Keio University School of Medicine, Shinanomachi 35, Shinjuku-ku, Tokyo 160-8582, Japan; ^2^Department of Emergency and Critical Care Medicine, Saiseikai Fukuoka General Hospital, 1-3-46 Tenjin, Chuo-ku, Fukuoka-shi, Fukuoka 810-0001, Japan

## Abstract

*Background*. Images of head CT for the supratentorial compartment are sometimes recommended to be reconstructed with a thickness of 8–10 mm to achieve lesion conspicuity. However, additional images of a thin slice may not be routinely provided for patients with trauma in the emergency room (ER). We investigated the diagnostic sensitivity of a head CT, where axial images were 10 mm thick slices, in cases of linear skull fractures.* Methods*. Two trauma surgeons retrospectively reviewed head CT with 10 mm slices and skull X-rays of patients admitted to the ER that were diagnosed with a linear skull fracture. All patients had undergone both head CT and skull X-rays (*n* = 410).* Result*. The diagnostic sensitivity of head CT with a thickness of sequential 10 mm was 89% for all linear skull fractures but only 56% for horizontal fractures. This CT technique with 10 mm slices missed 6% of patients with linear skull fractures. False-negative diagnoses were significantly more frequent for older (≥55 years) than for young (<15 years) individuals (*p* = 0.048).* Conclusions*. A routine head CT of the supratentorial region for patients in the ER with head injuries requires both thick-slice images to visualize cerebral hemispheres and thin-slice images to detect skull fractures of the cranial vault.

## 1. Introduction

Head injury is one of the most frequent reasons for visiting hospital emergency rooms (ERs) or clinics [1]. In this era of CT, most clinicians prefer head CT to skull X-rays for a radiological examination of head injury [2]. Thus, head CT is the first and, often, the only imaging study performed for head injuries in the ER. Recently, in many centres, CT images of the head have been reconstructed with a thickness of sequential 5 mm, but some neuroradiologists and neurologists recommend thicker slice thickness, of 8–10 mm, for the supratentorial compartment, to achieve lesion conspicuity. As a radiological examination of head injury in the ER, thin-slice CT images may not be routinely provided for detecting a skull fracture.

In the current study, we retrospectively examined historical data from 410 cases diagnosed with a linear skull fracture, based on skull X-rays and/or head CT performed at 10 mm thick slices. We showed that CT with a thickness of 10 mm missed 6% of patients admitted to the ER with linear skull fractures.

## 2. Methods

This retrospective observational study included patients admitted to the ER with linear skull fractures, diagnosed with skull X-rays and/or head CT sliced axially with a thickness of sequential 10 mm. We used a historical database that was obtained between January 2001 and September 2005, which had been anonymized and unlinkable to the personal records. All patients with head injuries that were referred to receive head CT during this period also underwent three-directional skull X-rays (anteroposterior, lateral, and Towne views). All data were acquired from the ERs of the following hospitals: Saiseikai Fukuoka General Hospital, Saiseikai Shizuoka General Hospital, Saiseikai Utsunomiya Hospital, Saiseikai Kanagawa Hospital, Saiseikai Kumamoto Hospital, and Saiseikai Mito Hospital. A skull fracture was diagnosed by board-certified neurosurgeons or consultant diagnostic radiologists, based on either the head CT or the skull X-rays. A total of 459 linear fracture lines were counted in 410 patients; 47 had two linear fracture lines and one had three fracture lines. In 90 cases, a linear fracture was accompanied by a depressed fracture, and all depressed fractures could be detected on both the CT and the X-rays.

Head CT images were reconstructed with a thickness of sequential 10 mm, brain window levels of 40–50, brain window widths of 100–150, bone window levels of 500, and bone window widths of 1600–4000. Different CT appliances were used at different hospitals. The Aquilion 4 (Toshiba Medical Systems, Tokyo, Japan) was used at Fukuoka, Kanagawa, Utsunomiya, Kumamoto, and Mito hospitals; the HiSpeed Advantage SG (General Electric, Fairfield, CT, USA) was used at Utsunomiya hospital; and the LightSpeed Plus (General Electric) was used at Shizuoka hospital.

For the present study, two trauma surgeons retrospectively reviewed the head CT and skull X-rays to detect fractures. At least one of the reviewers was board-certified by the Japanese Society of Acute Medicine. A skull fracture diagnosis was determined by consensus between the two reviewers, and disagreements were resolved with discussion. The fractures detected on head CT and/or skull X-rays were classified according to their location in one of four regions of the skull bone, frontal, parietal, occipital, or temporal bone, and, according to their orientation along the skull surface, defined as longitudinal (inclined 60°–90° from the orbitomeatal line), horizontal (inclined 0°–30° from the orbitomeatal line), or diagonal (inclined between 30° and 60° from the orbitomeatal line).

## 3. Results

The overall sensitivity of head CT was equivalent to that of skull X-rays for detecting a linear fracture (CT: 89% versus skull X-rays: 91%, Table 1). However, as expected, axial CT images acquired at 10 mm slices missed nearly half the fractures that were oriented in a horizontal direction (56% sensitivity), although they showed high sensitivity for longitudinal fractures (98% sensitivity). Three-directional skull X-rays showed nearly 90% sensitivity for detecting either longitudinal (88%) or horizontal (93%) linear fractures (Table 1).

No preponderance was observed for fracture location in any part of the skull bone among the 459 fractures studied (Table 2). However, fractures that were oriented horizontally, which were more difficult to diagnose based on 10 mm CT slices (Table 1), were observed most frequently in the parietal bone of the cranial vault (Table 2) shown in CT images of the supratentorial compartment.

In 24 out of 410 cases (6%), linear fractures were detected only on skull X-rays and not on head CT with 10 mm thick slices (Table 3). These false-negative skull fracture diagnoses based on CT were observed in older individuals (≥55 years) significantly more frequently than in young (<15 years) individuals (*Z* = 1.97, *p* = 0.048, Table 3).

## 4. Discussion

Recent advances in CT technology have enabled high resolution image reconstructions with data acquired at thin slices (0.5 to 1.5 mm), and helical CT scanning using thin slices is now routine practice in many countries including the UK, North America, and Japan. These data can be obtained with multidetector devices, and they provide superior diagnosis rates compared to skull X-rays [3]. Thus, skull X-rays have largely been replaced by helical scanning using thin slices, and patients are managed without the risk of additional irradiation that amounts to approximately 7 mGy with three-directional skull X-rays. A current protocol of head CT for patients with head injury in our hospital is that helical CT provides both images of thick (5 mm) and thin (1 or 1.25 mm) slices and bone surface images with volume rendering on request. Because thick- and thin-slice images are reconstructed by raw data obtained by 0.5 mm slice thickness, the radiation dose is not variable, for example, 72 mGy with 64-detector helical CT for adult head in our institution. However, thin-slice imaging and bone surface images are not routinely provided, because the majority of patients admitted to the ER that are referred for head CT have acute diseases, not traumas. Although 5 mm thick slices are widely used as the default setting, an 8–10 mm slice thickness has been recommended, particularly for detecting early ischemic lesions in the cerebral hemispheres (e.g., these CT scan settings were used in the Middle Cerebral Artery Embolism Local Fibrinolytic Intervention Trial Japan [4]). Thus, some institutions use head CT imaging protocols that specify 3 to 5 mm thick slices in the posterior fossa, to reduce artefacts at the skull base, but they specify 8 to 10 mm thick slices in the supratentorial compartment to achieve lesion conspicuity.

In the present study, among the 410 cases with linear skull fractures, we showed that CT with 10 mm thick slices missed 6% of the patients with linear skull fractures. A previous report analysed 39 patients with skull fractures and reported that 8% were invisible on CT performed at 1-cm intervals in the supratentorial portion; in contrast, all fractures could be detected on skull X-rays [5]. The false-negative rate of linear skull fracture detection with 5 mm CT thickness has not been reported, but bone window images of 10 mm thick slices, at least as shown in the present study, miss a significant percentage of skull fractures in patients admitted to the ER. Therefore, we recommend that patients with head injuries, including patients that possibly sustain traumas, should be screened with head CT with 5 mm (or larger) slices in the supratentorial compartment to examine the brain parenchyma and also CT with thin slices for detecting linear skull fractures of the cranial vault and skull base fractures as well.

Previous studies have elucidated the clinical variables that predict significant intracranial trauma. It is known that a skull fracture carries the highest relative risk when the patient displays factors in clinical examination or imaging that predict intracranial pathology [6]. It is well known that intracranial haemorrhage deteriorates with time, even when the head CT shows no distinct abnormalities immediately after an insult; this phenomenon is the so-called “talk and deteriorate” [7–9]. Therefore, it was recommended that patients with skull fractures should be closely observed at a hospital for 12 h or more after the insult [8, 9], even when their consciousness is not impaired at the initial assessment. In the ER, many patients with minor head injuries require consultation with emergency doctors. Previous CT imaging studies have revealed skull fractures in 3.8% of the patients in the ER that had minor head injuries rated 13 to 15 on the Glasgow Come Scale [10]. It is crucial to assess patients with minor head injuries appropriately with CT scans for both intracranial haemorrhagic lesions and skull fractures.

The present study showed that linear skull fractures of older patients (≥55 years) were missed more frequently than those of young patients (<15 years, Table 3) on head CT. Our data could not clarify why the CT sensitivity for a linear fracture was different for patients of different age groups. One possible explanation is that very fine fracture lines are likely to be obscured in CT images with thick slices and that fine fractures may appear more frequently in older than in younger individuals. This possibility is supported by the fact that, with age, the skull bone hardens and has less compliance; thus, it is more susceptible to cracking upon impact. In many countries, the proportion of older citizens is rising, and older individuals are susceptible to falling or slipping while walking. Consequently, increasing numbers of older patients appear in the ER with trauma. Therefore, patients with minor head injuries, particularly older individuals, should undergo both the routine CT imaging to visualize brain parenchyma and thin-slice images to detect linear skull fractures, for adequate predictions of intracranial haemorrhage. On the other hand, in pediatric head injuries CT scanning is not advocated in minor cases from the viewpoint of subsequent risk of malignancy [11]. Radiation exposure is approximately 40 mGy for children with a current 64-detector helical head CT. We should realize that use of CT scans in children to deliver cumulative doses of about 50~60 mGy might almost triple the risk of leukemia or brain cancer [11]. Thus, in pediatric practice, skull X-rays may still have a possible role to play in pediatric patients with minor head injuries.

## 5. Conclusions

This study showed that, among 410 patients admitted to the ER with a linear skull fracture, 6% of cases were missed on head CT with 10 mm thick slices. We concluded that thin-slice CT images are necessary in order to identify skull fracture. In pediatric practice, however, skull X-rays may be considered in those with minor head injuries.

## Figures and Tables

**Table 1:** Numbers of linear skull fractures and sensitivities of head CT with 10 mm thick slices and skull X-rays (anteroposterior, lateral, and Towne views).

Fracture orientation^*∗*^	Head CT			Skull X-rays			Head CT or skull X-rays
	True positive	False negative	Sensitivity	True positive	False negative	Sensitivity	True positive
Longitudinal	293	6	98%	263	36	88%	299
Horizontal	33	26	56%	55	4	93%	59
Diagonal	83	18	82%	98	3	97%	101
Total	409	50	89%	416	43	91%	459

^*∗*^Fracture orientations were determined with respect to the orbitomeatal line.

**Table 2:** The locations and orientations of linear fractures detected on head CT or skull X-rays.

Fracture orientation^*∗*^	Location in the skull bone				
	Frontal	Parietal	Occipital	Temporal	Total
Longitudinal	67 (74%)	37 (35%)	114 (93%)	81 (57%)	299 (65%)
Horizontal	6 (7%)	26 (25%)	8 (7%)	19 (13%)	59 (13%)
Diagonal	17 (19%)	43 (41%)	0 (0%)	41 (29%)	101 (22%)
Total	90 (100%)	106 (100%)	122 (100%)	141 (100%)	459 (100%)

^*∗*^Fracture orientations were determined with respect to the orbitomeatal line.

**Table 3:** Number of cases with linear fractures that were not detected on head CT with 10 mm thick slices.

	Head CT			Head CT or skull X-rays
	True positive	False negative	Sensitivity	True positive
<15 years	87	2	98%^*∗*^	89
≥55 years	172	16	91%^*∗*^	188
Total	386	24	94%	410

^*∗*^ *p* = 0.048 in <15 years versus ≥55 years by the test for the population proportion.
